# Distortions of Subjective Time Perception Within and Across Senses

**DOI:** 10.1371/journal.pone.0001437

**Published:** 2008-01-16

**Authors:** Virginie van Wassenhove, Dean V. Buonomano, Shinsuke Shimojo, Ladan Shams

**Affiliations:** 1 Division of Biology, California Institute of Technology, Pasadena, California, United States of America; 2 Department of Psychology, University of California at Los Angeles, Los Angeles, California, United States of America; 3 Department of Neurobiology, University of California at Los Angeles, Los Angeles, California, United States of America; Baylor College of Medicine, United States of America

## Abstract

**Background:**

The ability to estimate the passage of time is of fundamental importance for perceptual and cognitive processes. One experience of time is the perception of duration, which is not isomorphic to physical duration and can be distorted by a number of factors. Yet, the critical features generating these perceptual shifts in subjective duration are not understood.

**Methodology/Findings:**

We used prospective duration judgments within and across sensory modalities to examine the effect of stimulus predictability and feature change on the perception of duration. First, we found robust distortions of perceived duration in auditory, visual and auditory-visual presentations despite the predictability of the feature changes in the stimuli. For example, a looming disc embedded in a series of steady discs led to time dilation, whereas a steady disc embedded in a series of looming discs led to time compression. Second, we addressed whether visual (auditory) inputs could alter the perception of duration of auditory (visual) inputs. When participants were presented with incongruent audio-visual stimuli, the perceived duration of auditory events could be shortened or lengthened by the presence of conflicting visual information; however, the perceived duration of visual events was seldom distorted by the presence of auditory information and was never perceived shorter than their actual durations.

**Conclusions/Significance:**

These results support the existence of multisensory interactions in the perception of duration and, importantly, suggest that vision can modify auditory temporal perception in a pure timing task. Insofar as distortions in subjective duration can neither be accounted for by the unpredictability of an auditory, visual or auditory-visual event, we propose that it is the intrinsic features of the stimulus that critically affect subjective time distortions.

## Introduction

Subjective time is not isomorphic to physical time [1]: the subjective duration of an event can be systematically overestimated, a phenomenon referred to as “time dilation”, “time subjective expansion” [2] or “chronostasis” [3], [4]. Time dilation was recently proposed to rely on the predictability of the event to be judged: low probability events (i.e. high unpredictability) would be experienced as longer than high probability (i.e. high predictability) events of equal physical duration [2]. Distortions of subjective duration have also been reported in different contexts, namely, at the time of saccade [5], [6] or during voluntary action [7]. An extensive literature shows that the duration of an event is not solely experienced on the basis of its temporal properties: attentional, arousal and emotional levels, expectancy and stimulus context can all affect the experience of time [8], [9], [10]. Additionally, the time scale of the stimulus and the task used to measure participants' subjective duration have a bearing on the neural mechanisms involved in temporal processing [11], [12], [13], [14]. In the milliseconds to seconds range, time is perceived as a ‘subjective present’ (vs. ‘time estimation’) which inherently affects the perceptual structuring of the world [11], [13], [15] and thus provides crucial insights on perception. In a prospective (vs. retrospective) duration task, participants know prior to the experiment that they will report the duration of events, hence focusing the subject on the temporal properties of the stimuli [16]. Here, we tested duration perception of *sub-second* range (∼500 milliseconds) and highly ‘predictable’ auditory, visual, and auditory-visual events using prospective judgments.

Earlier studies have shown subjective time distortions in auditory [3], visual [2], [5], [6], [17], [18] and tactile [4], [7] sensory modalities but none has yet explored whether stimuli presented in one sensory modality could affect duration judgments in another sensory modality. Investigating cross-modal effects in time perception is crucial for determining whether time processes are centralized or distributed. The observation of time dilation effects in different sensory modalities has been taken as evidence for the existence of a common sensory-independent internal timer in subjective time perception [2], [3] but this is only a conjecture since similar results could be obtained if independent timers were to co-exist in each sensory modality. The dominant model of time measurement in the brain is the internal clock model. In its simplest form, an internal clock consists of a pacemaker which generates discrete events at a fixed frequency and an accumulator which counts these events; the resulting count can be compared with a duration stored in memory [13], [14], [19], [20]. In an amodal (sensory-independent or ‘supramodal’) clock model, the experience of time is mediated by a single pacemaker receiving inputs from any sensory modality. In the modality-specific or ‘modal’ view, each sensory modality has its own pacemaker leading to a distributed processing of temporal information[14], [21] (see Figure S1 in Supplementary Material, for a schematic rendering of internal clocks). Studies comparing the perception of duration across sensory modalities have shown that the duration of an auditory interval is often judged as longer than the same interval presented in the visual sensory modality [22], [23], [24]. These observations have lead to two specific (but non-exclusive) hypotheses with respect to clock models: (i) the latency of the on/off switch from the pacemaker to the accumulator may be more stable for the auditory than for the visual sensory modality and (ii) the rate of the pacemaker for the auditory inputs may run faster than for the visual ones [24], [25], [26]. However, these inter-sensory differences can also be accounted for by a distributed modal clock (e.g. modality-specific pacemakers and accumulators). The issue of a centralized vs. a distributed timing mechanism is complicated by discrepant findings: the improvements obtained by training participants on an auditory temporal discrimination task generalize to the tactile domain [27], to different frequencies [28], [29] and to different temporal tasks [30]. In vision, the perceptual improvements obtained after training on a visual temporal discrimination task transfer across hemispheres [31]. However, localized distortions of subjective time in vision have also been reported in adaptation experiments [32], [33]. Here, we thus examine the critical variables contributing to shifts in subjective time perception within and across the auditory and visual modalities and explore the notions of input predictability and intersensory interactions in the experience of duration.

**Figure 1:**
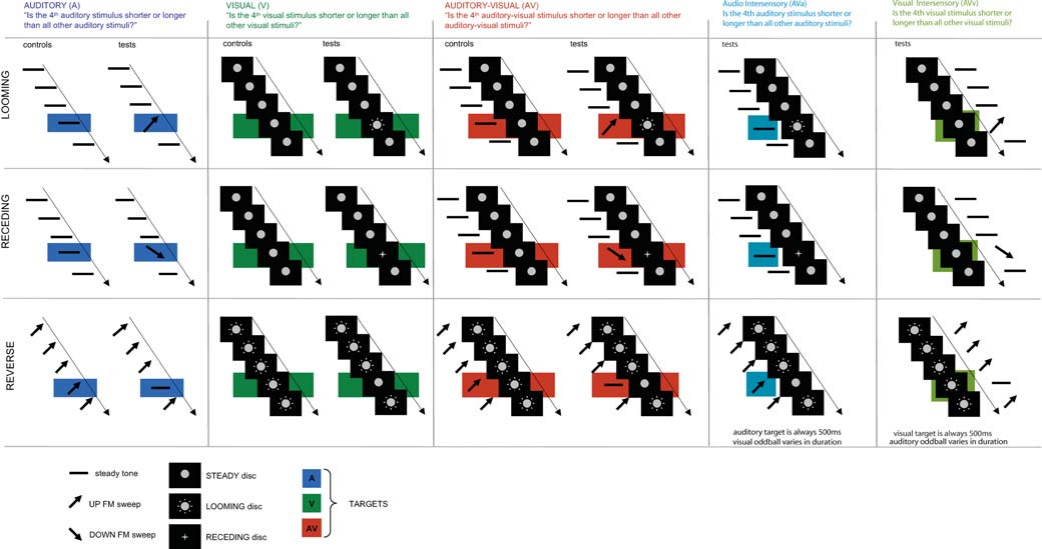
Experimental design. All Experiments tested unimodal (auditory only, first column, or visual only, second column), multisensory (congruent auditory-visual, third column) and incongruent or intersensory auditory-visual conditions (auditory intersensory, fourth column and visual intersensory fifth column). In the control conditions, the target (4^th^ stimulus in a stream of five stimuli) differed from the standards (stimulus 1, 2, 3 and 5; all 500 ms) in duration only. In the test conditions, the target differed from the standards in both feature and duration. In Experiment 1 (‘Loom’, first row) and Experiment 2 (‘Recede’, second row), the same control conditions were used, where standards were 500 ms discs or pure tones in visual and auditory displays, respectively. In the Loom tests, auditory standards were 500 ms pure tones, and auditory targets were upward going FM sweeps of varying duration; visual standards were 500 ms discs and visual targets were looming discs of different duration; auditory and visual conditions were combined in the multisensory condition. In the Recede tests, the target was a downward going FM sweep or a receding disc in the auditory and visual sensory modalities, respectively. In the control of Experiment 3 (‘Reverse’, third row), the auditory standards were upward FM sweeps and the visual standards were 500 ms looming discs. In the Reverse tests, the oddballs were a steady disc and a pure tone of variable duration in visual and auditory displays, respectively. The Loom, Recede and Reverse intersensory conditions consisted in presenting congruent auditory-visual standards but incongruent auditory-visual targets. An oddball was introduced in the sensory modality which was to be ignored. In the auditory intersensory conditions, participants evaluated the auditory target while neglecting visual inputs; conversely, in the visual intersensory conditions, participants evaluated the visual target while ignoring the auditory inputs. In the Loom auditory (first row, fourth column) and visual intersensory (first row, fifth column) conditions, the oddball was a looming disc and an upward FM sweep, respectively. In the Recede auditory (second row, fourth column) and visual intersensory (second row, fifth column) conditions, the oddball was a receding or a downward FM sweep, respectively. In the Reverse auditory (third row, fourth column) and visual intersensory (third row, fifth column) conditions, the oddball was a steady disc or a tone, respectively.

Auditory (A), visual (V), congruent (‘multisensory’) and incongruent (‘intersensory’) auditory-visual (AV) durations were tested in three experiments (see Figure 1). The main paradigm consisted in presenting five consecutive stimuli within a single trial: the standard stimuli (stimuli 1, 2, 3 and 5 in the stream) were 500 ms whereas the fourth stimulus (the target) varied in duration only (control conditions) or in both duration and in feature (test conditions). Participants were instructed prior to the start of each experimental block which sensory modality they should evaluate; each block consisted of either a control or a test condition in the A, V, congruent AV or incongruent AV presentations. In Experiments 1 and 2, the standards were 500 ms steady visual discs and/or auditory pure tones. In Experiment 1 (hereafter referred to as ‘Loom’), the target was a visual looming disc and/or an auditory upward frequency-modulated (FM) sweep whereas in Experiment 2 (‘Recede’), the target was a visual receding disc and/or an auditory downward FM sweep. In Experiment 3 (‘Reverse’), the standards were visual looming discs and/or upward FM sweeps while the target was a steady signal (visual disc and/or auditory pure tone). In all three experiments, intersensory conditions were introduced to test the effect of incongruent AV presentations on duration judgments of a target modality (i.e. A or V). The term ‘intersensory’ will henceforth be used to designate the *incongruent* conditions. In the ‘auditory intersensory’ conditions, participants reported whether the auditory target was shorter or longer than all other auditory stimuli in the trial; conversely, in the ‘visual intersensory’ conditions, participants judged whether the visual target was shorter or longer than all other visual stimuli in the trial. In the intersensory conditions, the target (always 500 ms in duration) was paired with an oddball (varying in feature and duration) in the modality which was to be ignored. Importantly, the target in the attended sensory modality was identical in all respects to the standards in the same modality. We will first discuss the results of the unisensory (A, V) and congruent multisensory (AV) conditions within each experiment and will then turn onto the results for incongruent presentations (intersensory conditions).

## Results

### Subjective time distortions in auditory, visual and congruent (multisensory) audiovisual displays

First, we tested our experimental design in A, V and congruent AV conditions: on a given trial, the target always occurred in 4^th^ position within a stream of four 500 ms standards. There was no element of surprise as to the (temporal or spatial) position of the oddball. Participants judged whether the target was “shorter” or “longer” than all other standards in the trial. In the Loom experiment, targets were looming visual signals and/or upward auditory FM sweeps; in the Recede experiment, targets were visual receding signals and/or downward auditory FM sweeps. In the control conditions, the target solely changed in duration whereas in the test conditions, the target changed in both feature and duration (see Figure 1, first and second row, respectively.)

**Figure 2:**
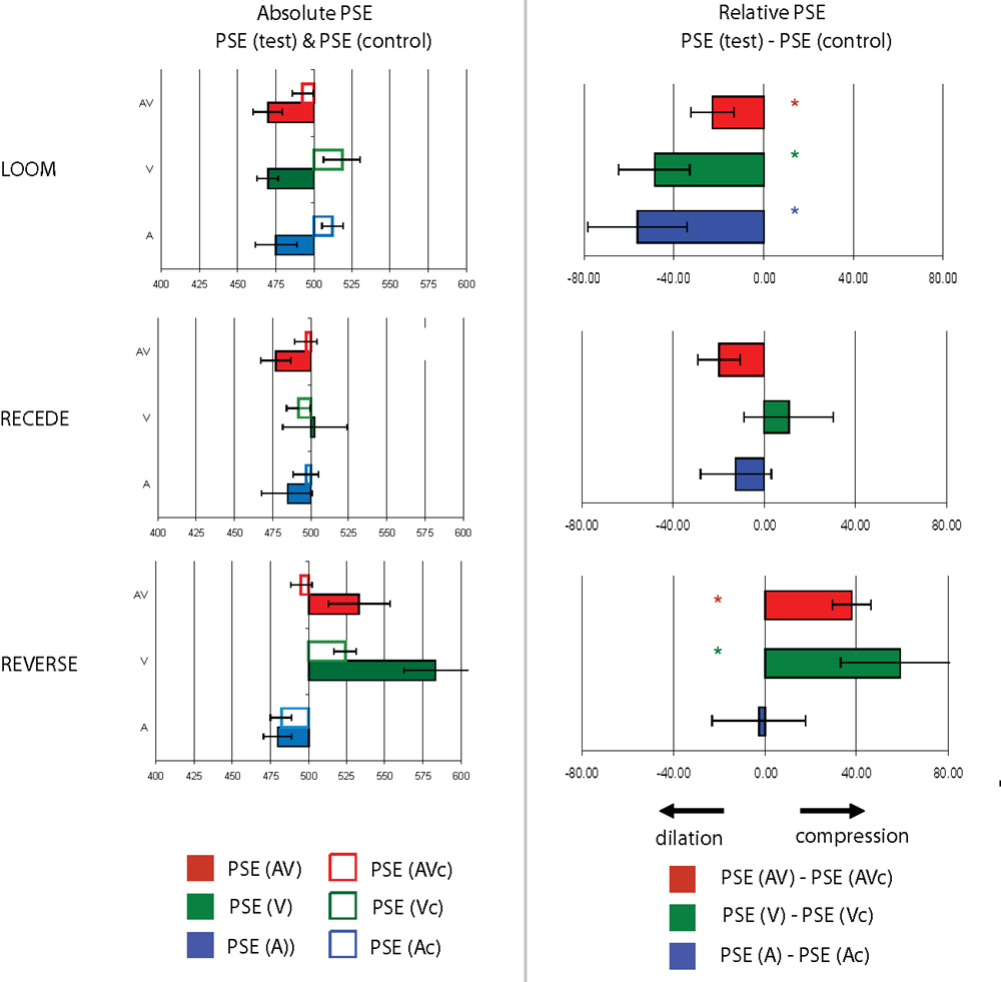
Subjective duration distortions in auditory, visual and congruent auditory-visual presentations. The points of subjective equality (PSE) were computed from the individuals' psychometric curves obtained in the control and test conditions. On the left hand-side, we report the obtained PSE for each experiment and auditory (blue), visual (green) and auditory-visual (red) conditions. On the right-hand side, we report the difference between the PSE obtained in a given test condition (e.g. visual test) and the PSE obtained in the associated control condition (e.g. visual control). In the relative PSE graphs, a positive shift of PSE indicates ‘subjective time compression’, thereby a given stimulus in the test condition is perceived as shorter than would actually be perceived by the participant in the control condition; conversely, a negative shift in PSE indicates ‘subjective time dilation’. Error bars are standard-errors of the mean. In the Loom experiment (first row), subjective time expansion is systematically observed in auditory (blue bar), visual (green bar) and congruent auditory-visual (red bar) presentations. In the Recede experiment (second row), no significant shift of PSE was observed. In the Reverse experiment (third row), both visual (green) and congruent auditory-visual (red) presentations led to a significant compression of subjective duration. No such effect was observed in the auditory (blue bar) condition. These results highlight both similarities and asymmetries in the distortion of subjective durations across sensory modalities.

In Loom, the points of subjective equality were derived from cumulative Gaussian fits of the individuals' percentage of longer responses for each condition (tests and controls in A, V and AV presentations). Figure S2 provides an example of an individual's psychometric fits in A, V and AV test and control presentations. The point of subjective equality (PSE) was defined for each individual as the duration corresponding to 50% of “longer” responses. Figure 2 provides the grand average of the individual PSE (left-hand side) together with the PSE differences between tests and controls obtained in each sensory modality and in each experiment (right hand-side). In Loom (Figure 2, first row), all three sensory modalities (A, V and AV) showed a significant decrease of PSE in the test conditions as compared to the control conditions. The decrease in PSE signifies that for an equivalent physical duration, a shorter looming (upward FM) signal was judged as longer than a steady disc (pure tone). A 3×2 repeated measures ANOVA with PSE as dependent variable and factors of modality (A, V and AV) and condition (test and control) showed a main effect of condition (F_1, 24_ = 21.091, p≤0.0001). No effect of modality (F_2, 48_ = 0.939, p = 0.393) or interaction of modality with condition (F_2, 48_ = 1.752, p = 0.184) were obtained, suggesting that the decrease of PSE was comparable across uni- and multi-sensory presentations. The temporal dilation effects observed in the Loom experiment were associated with a large effect size as evaluated by Cohen (d) and Hedge's (ĝ) indices (see Methods section): d = 0.77 and ĝ = 0.75 in the auditory conditions, d = 1.22 and ĝ = 1.18 in the visual conditions, and d = 0.62 and ĝ = 0.61 in the multisensory conditions.

This first set of results demonstrates that although participants could predict *when* and *which* oddball would occur in each experimental block and in each sensory modality of presentation, a significant subjective time dilation was observed in all conditions. The change in PSE could be due to (i) the predictability of feature changes in the target, (ii) the increased attention to the expected target, or (iii) the intrinsic properties of the stimuli. For instance, the increased perceived brightness (loudness) in the looming visual (auditory) target could relate to the experience of duration: intensity-duration dependency have seldom been studied but noted in both visual [34] and auditory contexts [35]. If such were the case, a stimulus with an identical rate of perceived brightness (loudness) *decrease* as that used in the looming signals of Experiment 1 should induce a comparable *increase* of PSE (i.e. a subjective compression of time in the same order of magnitude). This was tested in the Recede experiment, where oddballs were visual receding signals and/or downward auditory FM sweeps. An analysis of PSE similar to that conducted in the Loom experiment is reported in Figure 2b, where no change of PSE was observed. A 3×2 repeated measures ANOVA with PSE as dependent variable and with factors of modality (A, V and AV) and condition (test and control) confirm this observation: neither condition (F_1, 14_ = 0.133, p = 0.126), nor modality (F_2, 28_ = 2.234, p = 0.721) nor their interaction (F_2, 28_ = 1.34, p = 0.278) showed a significant effect.

The results obtained in the Loom and the Recede experiments indicate that although looming and receding signals provide an identical temporal rate with inverse directionality (i.e. increase/decrease in perceived brightness/loudness), they do not yield similar perceptual effects. While the former elicited time dilation in all sensory modalities (A, V and AV), the latter did not induce robust changes of duration. Hence, changes in perceived brightness or loudness cannot solely account for the observed changes in PSE. In contrast, an increase in perceived brightness/loudness may also increase the salience of the stimuli: auditory and visual looming signals are ecologically relevant because they signal approaching objects (and imminent collision) across many species [36], [37], [38]. Looming signals are salient and more attention-grabbing (exogenous attention) than other types of signals including the receding ones that were used here [39], [40]. A decrease in perceived brightness/loudness may thus also decrease the salience of the stimulus, leading the following conflicting result: a change in perceived brightness/loudness may draw attention to the target, while the directionality of the change (here, decrease) may lead to a decrease in the salience of the target. The tension between increased salience due to changing stimuli and the decreased salience due to the directionality of the change may have lead to the null result observed here. We next address whether salient standards such as looming-stimuli would induce a distortion of perceived duration in a steady target. Specifically, we predicted that the PSE to a steady target should increase i.e. that the subjective duration of the steady target embedded in a looming stream would be shortened.

### Induced subjective time compression

In the Reverse experiment, the standards were looming visual signals and/or upward auditory FM sweeps, whereas the target was a steady visual disc and/or an auditory pure tone (Figure 1, bottom row). As in Experiment 1 and 2, individuals' PSE were computed for each experimental condition. Figure 2 (bottom row) reports the grand average absolute PSE (left-hand side) and differences in PSE (right-hand side) for each sensory modality. A 3×2 repeated measures ANOVA with PSE as dependent variable, and with factors of modality (A, V and AV) and condition (test and control) was performed. Main effects of modality (F_2, 34_ = 29.697, p≤0.0001), condition (F_1, 17_ = 13.241, p≤0.002) and their interaction were found to be significant (F_2, 34_ = 7.149, p≤0.003). A paired t-test comparison of PSE between controls and tests showed that whereas a significant increase of PSE was observed in the visual (t_1, 34_ = 2.032, p≤0.001) and auditory-visual (t_1, 34_ = 2.032, p≤0.0001) conditions, no significant effect was observed in the auditory condition (t_1, 34_ = 2.032, p = 0.47). Large effect sizes were observed in the auditory (d = −1.01 and ĝ = −1.35) and auditory-visual (d = −0.69 and ĝ = −0.93) conditions.

Therefore, looming standards lead to the *compression* of subjective duration of a steady visual and auditory-visual target but not of an auditory target. Under the hypothesis of the salience effect discussed above, the target in the Reverse condition could either be experienced as ‘less salient’ as compared to the looming standards, or ‘more salient’ because it differs from the sequence of standard stimuli. The compression of subjective duration observed in the visual and auditory-visual conditions is more consistent with a *decrease* in the salience of the visual target induced by an increase of salience in the standards (i.e. looming is more salient than a steady target overall). In the auditory domain however, both decrease and increase in salience may be relevant leading to a null effect.

### Intersensory effects in experiencing duration

Thus far, we reported results in which the auditory and visual sensory modalities were tested separately or in congruent conditions i.e. when both modalities conveyed congruent temporal and feature information. Next, we examine the intersensory conditions, in which auditory and visual signals convey conflicting temporal and/or feature information. In these intersensory tasks, the standards (500 ms) and the targets were always co-occurring AV stimuli (Figure 1, fourth and fifth columns). In the Loom intersensory conditions (Figure 1, first row), the auditory (visual) target remained identical to the standard auditory (visual) stimuli (500 ms tone or steady disc) but was paired with a looming visual disc (upward auditory FM sweep) of variable duration. In the Recede experiment (Figure 1, second row), the auditory (visual) target was paired with a receding disc (downward FM sweep). In the Reverse experiment (Figure 1, third row), the auditory (visual) target was paired with a steady disc (tone) of variable duration. The results for all three experiments are now grouped as a function of the intersensory condition of interest, namely, the effect of audition on visual duration (visual intersensory tasks, ‘AVv’) and the effect of vision on auditory duration (auditory intersensory tasks, ‘AVa’).

#### Visual intersensory conditions: auditory duration seldom captures visual duration

**Figure 3:**
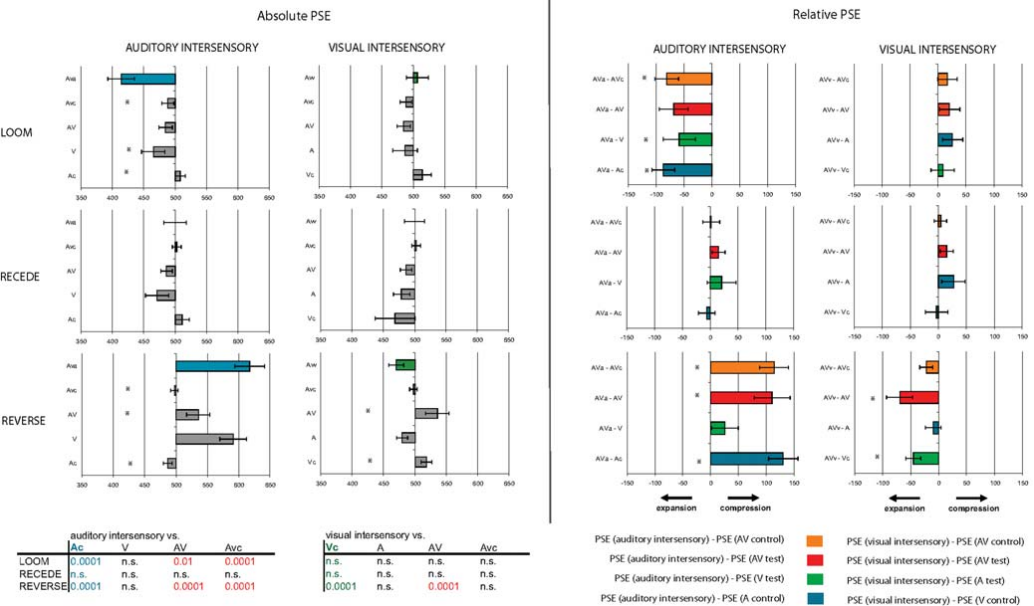
Subjective duration distortions in intersensory conditions (incongruent auditory-visual presentations). The PSE for the auditory (blue) intersensory conditions and the visual (green) intersensory conditions and their relevant control conditions (gray) are reported in the left panel as ‘absolute PSE’. The PSE differences between the intersensory conditions and their possible controls are reported in the right panels as ‘Relative PSE’. The corrected t-tests values are reported in the adjacent table for auditory and visual intersensory conditions in all three Experiments. The PSE obtained in a given intersensory (e.g. auditory intersensory) condition could be compared with (i) the auditory control (Ac), (ii) the visual test (V), (iii) the auditory-visual test (AV) or (iii) the auditory-visual control (AVc). A positive shift of PSE indicates ‘subjective time compression’ and a negative shift in PSE indicates ‘subjective time expansion’. Error bars are standard-errors of the mean. In Loom (first row), subjective time expansion is observed in the auditory intersensory condition when compared to the unisensory presentations (Ac, blue and V, green) and the congruent AV test (red); in the visual intersensory condition, no effect was observed suggesting that vision captures auditory duration but not the opposite. In Recede (second row), no significant intersensory effects were observed in either auditory or visual intersensory conditions. In Reverse (third row), the visual oddball captures auditory duration towards compression (blue bar) whereas the auditory oddball captures visual duration towards expansion (green bar). The auditory intersensory condition significantly differed from Ac, AV and AVc; the visual intersensory condition significantly differed from Vc and AV. These results provide evidence that visual information influences auditory temporal perception, but that the converse is surprisingly seldom observed.

The PSE quantification obtained in the visual intersensory conditions are reported in Figure 3: the absolute PSE are reported in the second column and the relative PSE, in the fourth column. In the Loom experiment (Figure 3, first row), the PSE obtained in the visual intersensory condition (second and fourth column) did not significantly differ from the visual control (green) or the auditory test (blue) conditions. No significant difference was observed between the PSE obtained in the visual intersensory condition and the AV test (red) or control (orange) conditions. Thus, the looming auditory event did not induce temporal dilation of visual duration in this task, which is particularly surprising given the robustness of subjective duration dilation observed in the auditory alone condition. In the Recede experiment (Figure 3, second row), a similar profile is observed (second and fourth column): auditory information does not significantly shift the visual PSE when compared to the visual control condition (green) and the multisensory test and control conditions (red and orange, respectively). This result is consistent with the lack of time distortion observed in the A, V and congruent AV conditions. In the Reverse experiment (Figure 3, bottom row), the PSE obtained in the visual intersensory condition show a significant time dilation effect with respect to the visual control condition (green) (t_1,20_ = 2.085, p≤0.01; effect sizes: d = −0.43 and ĝ = −1.15) and the multisensory test (red, t_1,20_ = 2.085, p≤0.009; effect sizes: d = −0.43 and ĝ = −1.18.)

Altogether in the visual intersensory conditions, auditory information captures subjective visual duration only in the Reverse experiment. This result is intriguing considering (i) that no distortion in duration was observed in the Reverse auditory test for a steady target and (ii) that the direction of PSE shift would be expected to be towards duration compression. One possible explanation is that even though participants were instructed to ignore the sound, they could not ignore it. Judging visual duration while paying attention to the sound may have caused a *contrast effect* across sensory modalities resulting in the dilation of perceived visual duration in the Reverse condition. However, it is unclear why a contrast effect would selectively operate in the Reverse condition but not, for instance, in the intersensory Loom condition.

#### Auditory intersensory conditions: visual capture of subjective auditory duration

In the Loom experiment (Figure 3, first row), a significant negative shift of PSE in the intersensory auditory condition was observed when compared to the auditory control (t_1,11_ = 2.08, p≤0.0003; d = −0.58 and ĝ = −1.58), the multisensory test (t_1,10_ = 2.1, p≤0.01; d = −0.4 and ĝ = −1.17 ) and the multisensory control (t_1,11_ = 2.08, p≤0.001; d = −0.54 and ĝ = −1.48) conditions. Visual inputs capture auditory duration with time dilation. In the Recede experiment (Figure 3b, blue bar), no significant shift of auditory intersensory PSE was observed as compared to the control condition (t_1,10_ = 2.1, p = 0.96). Here, a visual receding signal does *not* alter the auditory point of subjective equality. This result is again consistent with the lack of temporal distortion obtained in the V, A, and congruent AV conditions. In the Reverse experiment (Figure 3c, blue bar), a visual oddball affected subjective auditory duration with a significant positive change of PSE when compared to the auditory control (t_1,12_ = 2.07, p≤0.0001; d = 0.63 and ĝ = 1.84), the multisensory test (t_1,12_ = 2.18, p≤0.01; d = 0.57 and ĝ = 1.52) and the multisensory control (t_1,18_ = 2.1, p≤0.001; d = 0.6 and ĝ = 1.62). In the Reverse Experiment, time compression is induced in the auditory modality via visual presentation but no time compression was observed for the auditory alone condition. Further discussion of this effect is provided in the next section.

### Auditory-visual integration and perceived duration

**Figure 4:**
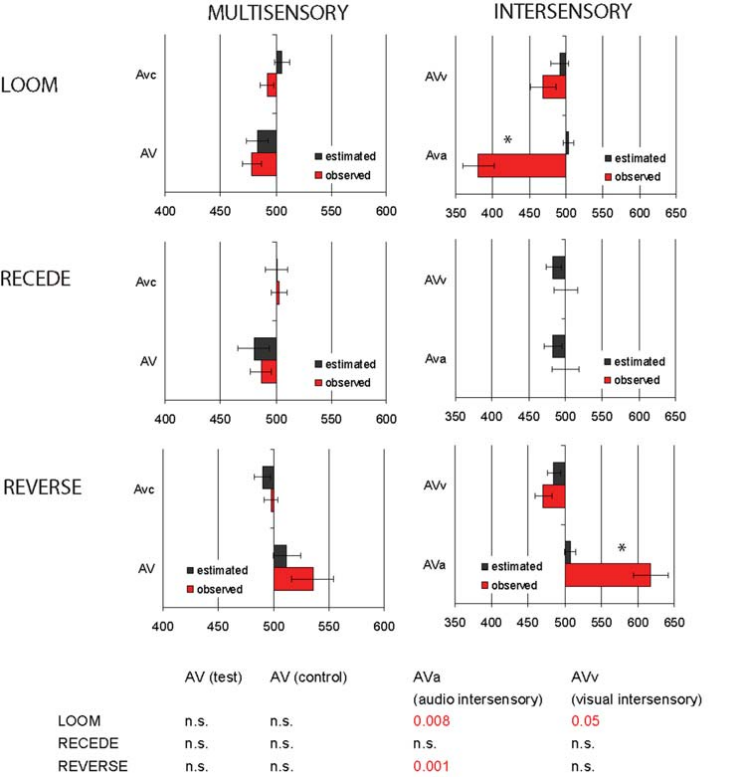
Forced-fusion model: comparison between predicted and observed PSE in congruent and incongruent auditory-visual presentations. In each graph, the black bars indicate the “estimated”, and the red bar, the “observed” PSE. The adjacent table reports the results of paired t-tests between predicted and observed PSE. In the congruent AV presentations (left column) and for all three experiments, the predictions of the forced-fusion Bayesian model did not significantly differ from the observed PSE. In the incongruent conditions (right column), the model fails to predict the perceptual outcomes observed in the auditory intersensory conditions of the Loom and the Reverse experiments. Additional comparisons between predicted and observed variances in these conditions are provided as Supplementary Material in Figure S3.

**Figure 5:**
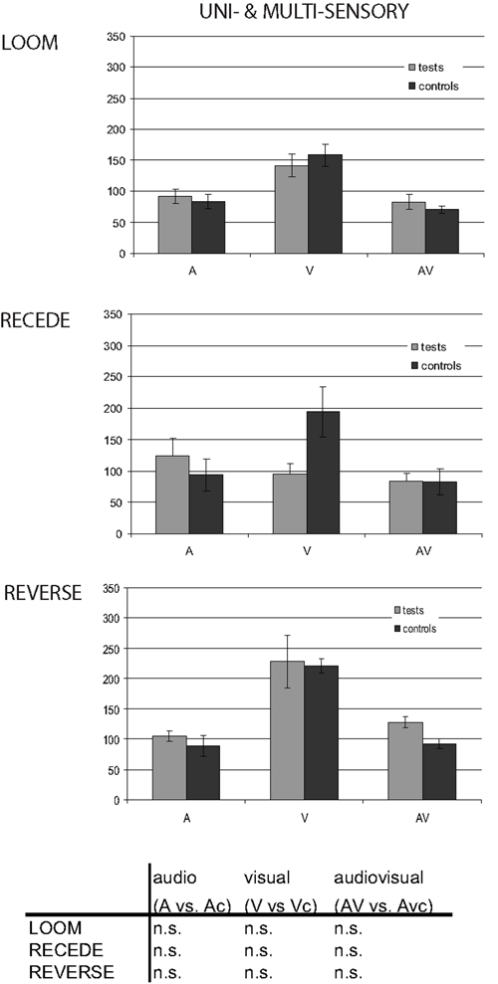
Variance in uni- and multi-sensory observed data. We report the variance for the tests (gray) and controls (black) of the auditory, visual and auditory-visual conditions in the Loom (top row), Recede (middle row) and Reverse (bottom row) experiments. No significant differences of variance were observed between tests and controls within each modality of presentation. Bars indicate standard-errors of the mean.

**Figure 6:**
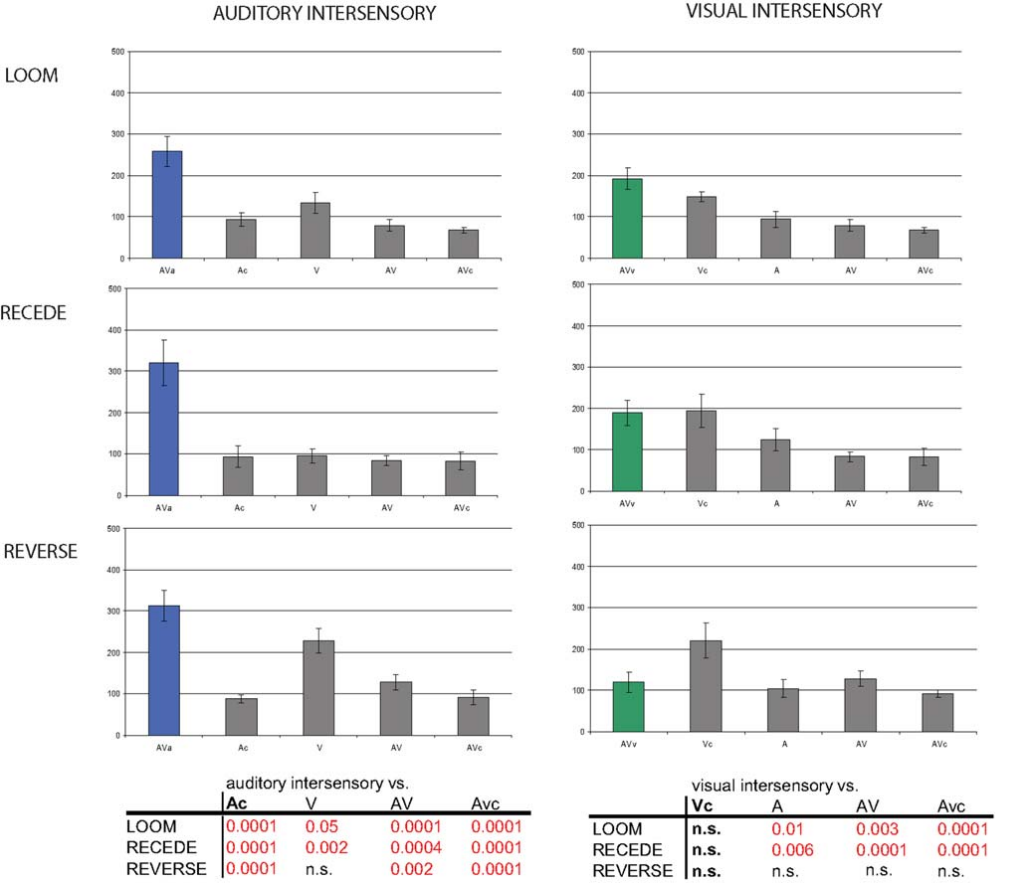
Variance in intersensory observed data. Variance for the auditory intersensory (blue, left column) and visual intersensory (green, right column) conditions are reported along with their respective control conditions (gray) in each experiment. The tables indicate the significant variance effects between the test and possible control conditions. A significant increase of variance was observed in the auditory intersensory conditions with respect to variance in auditory control, visual test, multisensory control and test conditions in all Experiments to the exception of the visual test in the Reverse condition. A significant increase of variance was observed in the visual intersensory conditions of the Loom and Recede experiments with respect to the auditory, multisensory test and control. In all experiments, no difference was observed between the visual intersensory and the visual control conditions and all possible control conditions in the Reverse experiment. Bars indicate standard-errors of the mean.

The ‘modality appropriateness hypothesis’ [41] has long proposed that the more precise modality dominates the integration of a multisensory event: audition has often been referred to as the dominant channel in temporal tasks [42], [43], [44] and visual timing has been suggested to be encoded in an auditory form [45]. While providing a useful theoretical framework for multisensory integration, the modality appropriateness hypothesis does not provide a quantitative account of multisensory perceptual effects. More recently, Bayesian models have successfully accounted for multisensory integration in a variety of contexts [46], [47], [48], [49], [50]. We here compare the predictions of a traditional model of multisensory integration [47], [51] with our data on the perception of multi- and inter-sensory AV durations. We refer to this model as “forced-fusion” as it assumes that the signals of the different sensory modalities are always completely fused into a single percept (see [46], [52] for discussion). In order to compare the observed data with the predictions of the traditional forced-fusion model, we used a method similar to the one described by Alais and Burr [53]. In Figure 4, we report the predicted PSE in the multisensory (congruent) or intersensory (incongruent) AV conditions (black bars) based on the independent combination of the PSE obtained in each sensory modality (A and V alone) and in each condition (control or test), and the estimated weight of each sensory modality. The red bars denote the observed PSE in each experiment. The outcomes of two-tailed paired t-tests between the predicted and observed measures across participants are reported in the table of Figure 4. As can be seen, the forced-fusion model predicted the observed data well when auditory and visual stimuli were congruent i.e. in the multisensory conditions. However, this model failed half of the time in predicting the direction of PSE shift when auditory and visual durations were incongruent, in particular under the auditory intersensory conditions of the Loom (t_1,16_ = 2.1, p≤0.008) and Reverse (t_1,20_ = 2.08, p≤0.001) experiments. In the auditory alone condition of the Reverse experiment, no distortion of subjective duration was found, yet the presentation of incongruent visual information during the auditory presentation compressed the perception of auditory duration. This finding cannot be accounted for by a forced-fusion model. Additional comparisons between the model predictions and the observed variance of the multisensory and intersensory conditions are provided in Figure S3. Note that all observed variances are reported in Figure 5 and 6. In Figure S3, we report the comparisons between the observed and the predicted variances in multi- and inter-sensory conditions. While the model accounts well for the observed variance in the multisensory conditions, it largely underestimates the variance observed in the intersensory conditions - with the exception of the Reverse visual intersensory condition.

In the visual intersensory condition, we observed no significant difference of PSE between the auditory control and the visual intersensory condition (leading to a significant dilation of duration). One possible explanation for this result is the observation that the absolute auditory control PSE in the Reverse conditions is significantly smaller than those observed in the visual control condition (t_1,22_ = 2.07, p≤0.001) (see Figure 2, bottom row). This comparison is in line with prior observations suggesting that for the same physical duration, the auditory is judged as longer than the visual stimulus [24]. In the intersensory presentation then, the auditory stimulus captures the duration of the visual stimuli. The result for this condition is consistent with (i) no variance change in visual intersensory condition (Figure 5) and (ii) the model prediction of the PSE change (Figure 4). In the auditory intersensory condition, a compression of duration was observed and as can be seen in Figure 6, an increase of variance was observed that did not significantly differ from that observed in the visual test condition. In this case, the forced-fusion model does not predict the change of PSE (Figure 4) nor the increase in variance (Figure S3). Both auditory and visual intersensory conditions of the Reverse experiment illustrate cases of intersensory captures in duration judgment. In the auditory intersensory case, the less variable sensory modality is not the most influential in the decision process, suggesting that some other factors may be at work. One possible explanation would be the existence of a multisensory contrast effect in which conflicting duration information presented in two sensory modalities is magnified when reporting the perceived duration of only one sensory modality. This hypothesis will require further testing as it is not entirely consistent with the results observed in the Loom condition. Altogether, these results show that at the time scale of a few hundreds of milliseconds, the temporal cues provided in the visual channel can compromise the temporal experience of an auditory event and that the auditory sensory modality may not always be the privileged channel in the experience of duration.

## Discussion

Subjective time dilation was consistently found in auditory, visual and auditory-visual presentations for a visual stimulus increasing in size, and an auditory event increasing in frequency (Loom experiment). These results establish that the subjective dilation of perceived duration occurs even when the target is predicted and expected. Second, a decrease in visual size and auditory frequency (Recede experiment) did not lead to significant distortions of subjective time, suggesting that orienting attention to the duration of the odd stimulus is not necessary to produce a subjective expansion of time; rather, the very fact that subjective time dilation was selective to the looming signals suggest that the salience of these stimuli is a major feature in the subjective experience of time. In the Reverse experiment (looming standards, steady target), we observed a robust compression of subjective duration in visual and auditory-visual but not in auditory presentations; these results further highlight the role of *contextual salience* in the experience of time, at least in vision. Here, the degree to which a target is salient may be a combination of (i) the ecological value of a stimulus (e.g. looming equals ‘approaching object’) and (ii) the temporal context within which the stimulus is embedded. If oddball-ness was the sole factor in orienting attention to the target, a dilation of subjective duration should always be observed in our conditions because the target always differed from the standards in features and/or duration; this is not what we observed in the Recede and Reverse experiments, suggesting that it is the salience of the target that matters. With respect to multisensory integration in duration perception, our results show asymmetries within and across sensory modalities. Visual inputs robustly lengthened and shortened the experience of duration in audition (Loom and Reverse experiments, respectively) whereas auditory inputs seldom lengthened visual subjective duration (Reverse experiment). The influence of vision on the subjective duration of auditory events is not straightforwardly accounted by a ‘forced-fusion’ model of multisensory integration as will be discussed below.

In the current experiments, the target was always presented in 4^th^ position and at the same location in the stream of standard events. In the test blocks, the probability of a feature change in the target was also constant across trials (i.e. equal to one), leaving the duration as the sole unpredictable variable. Nevertheless, our data show a robust dilation of subjective time which replicates prior studies that have used unpredictable targets [2]. In internal clock models, prospective duration tasks have been proposed to rely heavily on attentional resources [16]: the participant's state of arousal affects the *rate* of the pacemaker(s) whereas attention affects the *latency* of the switch to the accumulator i.e. the onset of the time keeper [22]. Therefore, a shift of attention to a target stimulus could lead to an early opening of the switch, in turn leading to a lengthening of the experienced duration (see Figure S1). Other studies have suggested that the auditory switch may be more stable than the visual switch [24], which would lead to greater variability in visual time keeping than in auditory time keeping [27]. Our analyses of variance (Figure 4 and 5) show a tendency for visual conditions to be of equal or more variability than the auditory conditions, supporting the notion that auditory and visual time keeping mechanisms are not entirely shared and ultimately, that sensory-specific properties are preserved in the extraction of temporal cues. Under the accumulator/switch framework, the distortions of time we observed could thus be interpreted as follows: dilation and compression of subjective duration entails a faster and slower rate of the pacemaker, and/or a shorter and longer latency of the switch, respectively. While reasonably fitting the looming (‘arousing’ stimulus) and the receding (‘non-arousing’) data, the problem emerges for the results obtained in the Reverse experiment and in particular, it is unclear why (i) a non-arousing steady stimulus would lead to compression in vision but not in audition, and (ii) why a shift of attention would occur much later in vision than in audition. Additionally, the observed variability in the auditory intersensory judgments is superior to that of the visual intersensory judgments (Figure 6). Under the accumulator/switch framework, one would needs to posit that visual (auditory) inputs can change the latency of the auditory (visual) switch or the rate of the auditory (visual) pacemaker to explain these changes in variability. Our data are thus difficult to interpret within this framework, and offer new challenges for the internal clock model.

Numerous stimulus attributes can clearly affect duration estimation [2]–[14]. Here, our goal was to minimize the effect of attentional orienting by providing consistent trials within which one main factor would vary, namely, the properties of the target in feature or duration space. An attentional account for the dilation of subjective time was previously formulated by Tse and colleagues [2]. Here, we refine this suggestion by showing that the salience of a target with respect to a stream of standard events - independently of whether the target is expected or not - is a determining factor for subjective distortions of time perception. Here, it is argued that the unpredictability of a target is unnecessary for temporal distortions but that it is nevertheless likely to influence time perception. For instance, in our Receding experiment, we observed no temporal distortion in contrast to the temporal dilation reported by Tse and colleagues [2] for a similar stimulus configuration. Again, a major difference between the two experiments is that of the uncertainty of the target. In [2], the receding stimulus is unpredictable and the dilation effect may be accounted for by its unpredictability; when this uncertainty is removed as in our Receding experiment, this stimulus does not induce time dilation. Additionally, when participants were asked to respond to all stimuli in the train (see Experiment 7 in [2]) the overall temporal dilation effect diminished suggesting a role for task-dependent attentional orientation in their experiments. One possibility is that uncertainty is a dominant factor relative to the salience of the stimulus in time dilation but when the unpredictability of the stimulus is removed, it is the sensory features that prevail, leading to different pattern of temporal distortion including time compression (see our Reverse Experiment). This interpretation converges with a recent study looking at the effect of stimulus predictability on duration judgments [18]. An additional component is the potential contribution of emotional valence as looming stimuli are ‘threat’ signals (i.e. negative emotional valence) [36]. Faces with a strong emotional valence have been shown to increase the perceived duration of the face presentation [54] although no duration dilation was found when comparing an arousing stimulus to a neutral stimulus in an oddball paradigm [18]. In one experiment, Tse et al. [2] used mannequin figures and showed an overall smaller temporal dilation for these stimuli. Among those stimuli that were less predictable, they showed larger temporal dilation effects, suggesting that there is an interaction between the ecological relevance (or the ‘semantics’ [2]) of the stimuli and their probability of occurrence.

Although our results do not directly address the neural mechanisms involved in subjective time perception, they are parsimonious with the notion that temporal processes below the second range are not centralized but are an inherent property of cortical networks [55], [56]. Traditional clock models do not differentiate (or seldom address the difference) between the supra- and sub-second range durations [14] but the hypothesis that temporal cues can be extracted locally - i.e. early in the hierarchy of the analytical sensory pathways - is more consistent with a sub-second range temporal processing model [56]. For short durations, recent findings indicate that the extraction of temporal cues such as visual temporal frequency is spatially confined [29], [32], [33], [56], [57]. Such results have led to the hypothesis that temporal processing could occur as early as V1, and that the neural mechanisms underlying time processing could be local [32], [33], [57].

A recent study comparing auditory and visual filled duration judgments and using combined magneto- and electro-encephalographic recordings shows an intricate pattern of transient and sustained activity in both sensory and non-sensory specific cortical areas [58]. Of particular interest, the authors report sensory-specific sustained responses which share the same cortical sources as the early sensory-specific transient responses. The authors also report a contingent-negative variation (CNV) which was independent of sensory modality, whose sources originated in a fronto-parietal network and which was concurrent with the sensory-specific sustained responses. These results suggest parallel ongoing temporal processing in sensory-specific pathways together with a component associated with the retention of information and working memory [59]. Other EEG studies also point out to an early differentiation of sensory-specific components that are tied to the duration of the stimuli with respect to the standards [60], further indicating local processing of temporal cues. Additionally, parietal areas (in particular, the right Inferior Parietal Lobule or IPL) have recently been argued to be part of a ‘when’ pathway [61] and activation of the IPL has indeed been reported during attentional orientation to time [62], [63] and multisensory temporal tasks [64]. Neurons in parietal areas show time-dependent firing properties [65] which converge with the notion that time may be encoded in a state-dependent network [56]. The IPL has also been categorized as a ‘metamodal’ (or amodal) area [66], providing a potentially crucial cortical area for the interactions of auditory and visual durations observed here.

With respect to the novel multisensory and intersensory effects reported here, the “modality appropriateness hypothesis” [41] argues that the most precise modality contributes most to the formation of a multisensory percept. Specifically, the temporal and spatial dimension would be dominated by the auditory and the visual sensory modalities, respectively. However, the assignment of sensory dominance may not always follow this strict dichotomy. The underlying assumption of the “modality appropriateness hypothesis” is that auditory temporal resolution is more precise than that of the visual modality. This assumption is based on prior studies of auditory-visual synchrony [42], [43], [44], but temporal simultaneity judgments do not entail the involvement of the temporal processing system in which the analysis of the time that has elapsed is needed [13], [21]. Time perception encompasses many processing levels (from the sub-second to years) that engage different brain mechanisms [11], [12], [13], [14], [15]. In synchrony studies (a scale of a few to tens of milliseconds) the auditory modality is likely to be more reliable than vision, with temporal rates of integration as fine as a couple of milliseconds [67], [68]. However, dynamic visual stimuli bear better temporal resolution than static ones [31] suggesting that dynamic visual events may have a comparable temporal resolution to that of auditory stimuli (e.g. temporal frequency in the 4–8Hz range has been defined as the limiting temporal factor in vision [57]). Our data show that audition may not always be the dominant channel for temporal information. The pattern of multisensory interactions found in this study appears inconsistent with the traditional forced-fusion model of multisensory integration: (i) the intersensory effects and (ii) the variance observed in both multi- and inter-sensory conditions are not well predicted by the model, suggesting that some stimuli properties need to be incorporated in the model (for instance, as priors). Future studies should investigate alternative models of multisensory perception [46], [69], [70] to examine whether models that do not *a priori* assume integration across sensory modalities can better account for multisensory interactions in time perception. Our results further suggest that duration judgments depend on the salience of the stimuli and not solely on the temporal cues afforded by each sensory modality. Previous studies have shown that contextual salience could alter visual perception when embedded in an auditory-visual context [71]. In the intersensory conditions, additional contextual cues may alter the duration of perception when combined across sensory modalities. In the Reverse auditory and visual intersensory conditions, opposite effects were found that could indicate a contrast mechanism in the estimation of duration between the two sensory modalities. In multisensory context then, a systematic mapping of unisensory *and* multisensory salience may help understand the specific contribution of each sensory modality to the representation of duration.

In summary, distortions in subjective temporal perception were found in auditory, visual and auditory-visual domains. The dilation and compression of subjective time were observed despite the predictability about *when* and *which* oddball would occur. The characteristics of distortion in subjective duration showed asymmetries across sensory modalities: vision captured audition in the experience of time while audition seldom influenced visual subjective duration. The pattern of results reported here is difficult to reconcile with our current understanding of duration perception and classic model of multisensory integration. Nevertheless, our results indicate that on a sub-second time scale, unpredictability is not the only factor that can produce shifts in subjective duration. We thus suggest that the contextual salience of the stimuli is a critical factor for the perception of duration at this time-scale, a feature that could be incorporated in models of multisensory and time perception.

## Materials and Methods

### Participants

A total of fifty-nine participants (34 females, mean age 22.1 years) took part in the study. Twenty-five participants (16 females, mean age 20.6 years) took part in Experiment 1, fourteen of whom were also tested on the intersensory conditions of Experiment 1. Fifteen participants (7 females, mean age 26.4 years) completed Experiment 2, and eighteen participants (8 females, mean age 20.7 years) completed Experiment 3. All participants were naïve to the purpose of the study and participated in only one experiment. All experiments were run in accordance with the University of California Human subjects guidelines and the Declaration of Helsinki.

### Stimuli

Visual stimuli consisted of a gray disk centered on the monitor screen and displayed on a black background. In the steady stimulus condition, the disk subtended two degrees of visual angle. The looming and the receding visual signals consisted of a centered gray disk changing in size from 2 to 5 degrees and from 5 to 2 degrees of visual angle, respectively. In the deviant stimuli, the change in size was constant regardless of the duration. The steady auditory stimuli consisted of a pure 1 kHz tone with 5 ms on/off linear ramp. The looming auditory signal consisted of an upward FM sweep centered at 1 kHz spanning a 500 Hz bandwidth (i.e. ranging from 0.75 to 1.25 kHz). The receding auditory signal consisted of a downward FM sweep centered at 1 kHz and ranging from 1.25 kHz to 0.75 kHz. Both looming and receding auditory signals were linearly ramped (on/off, 5 ms) and spanned the same initial and final frequency points regardless of signal duration. All stimuli were created using Matlab™ 7.1 (The Mathworks, Inc., Natick, MA) and presented in conjunction with the Psychophysics Toolbox extensions [72], [73] on a Mac G4 (Experiments 1 and 2, ‘Loom’ and ‘Recede’) or a Mac G5 (Experiment 3, ‘Reverse’).

All auditory, visual or auditory-visual standard stimuli were 500 milliseconds in duration. All auditory (A), visual (V) or auditory-visual (AV) oddballs were +/− 24%, +/− 10% or +/− 4% of the standard duration (i.e. 380 ms, 450 ms, 480 ms , 520 ms, 580 ms or 620 ms.) The inter-stimulus intervals (ISI) were pseudo-randomly chosen from 750 ms to 950 ms in steps of 20 ms. The randomization of the ISI was used to prevent participants from using rhythmic cues in their duration judgments. The inter-trial intervals lasted one second following participants' response.

In all experiments, each trial consisted of a train of five stimuli. This paradigm was designed in order to avoid possible confounds of stimulus position. Precisely, it has been reported that the first event in a train of visual stimuli tends to be judged as longer than all other subsequent events of equal duration [74]. For this reason, multiple standards were used in order to provide sufficient exemplars of the standard durations. Additionally, the fourth stimulus was always the target: participants judged whether the target was “shorter” or “longer” than all other stimuli in the trial (i.e. the first, second, third and fifth stimuli.) In the *test* conditions, the target differed from the standard stimuli in feature (e.g. if the standards were steady sounds, the target was a looming sound) and in duration (the standards were always 500 ms while the deviants took any of the deviant duration values described above). In the *control* conditions, the target only differed from the standards in duration (e.g. if all standards were steady, the target was also steady but changed in duration). The results from the control conditions provided a psychometric curve for the changes in stimulus duration alone allowing for an estimation of the true point-of-subjective equality for a 500 ms duration stimulus (as opposed to veridical duration.)

In all experiments, eight conditions were tested as follows: auditory test and auditory control, visual test and visual control, auditory-visual test and auditory-visual control, intersensory auditory test (visual deviant), intersensory visual test (auditory deviant). In the auditory-visual tests and controls, both auditory and visual stimuli had the same durations. Hence, in these multisensory conditions, both sensory modalities were congruent with respect to their duration. In the intersensory conditions, the auditory and visual stimuli differed in duration. In the intersensory auditory test, the auditory target was always 500 ms while the visual target (which was to be ignored) took any of the target durations described previously. Conversely, in the visual intersensory conditions, the visual target was always 500 ms while the simultaneously occuring auditory events took any of the target durations described above. Hence, in the intersensory conditions, the auditory and visual durations were incongruent. The order of presentation for all these conditions was pseudo-randomized across participants.

Auditory-visual stimuli were aligned to the millisecond using the audio card and a photo-detector connected to an oscilloscope for auditory-visual output signals alignments. In the intersensory conditions, where auditory and visual were incongruent in durations, the stimuli were aligned to their mid-duration point. For instance, if a 620 ms duration stimulus was paired with a 500 ms duration stimulus, the onset and offset of the longest stimuli started and ended 60 ms before and after the 500 ms duration stimulus, respectively.

### Procedure

All experiments took place in a dimly lighted room. Participants sat 57 cm away from the computer screen and stabilized their heads using a chin-rest. The auditory stimuli were delivered via loudspeakers placed on each side of the monitor screen and at the same height of the visual stimulus. The sound pressure level was set to 70 dB. The visual stimuli were delivered on 19” Cathode Ray Tube monitor with a refresh rate of 100 Hz. Prior to all experiments, participants were given a few practice trials on each experimental condition. In all experiments, an experimental block started with a statement specifying which sensory modality should be considered for the participant's duration judgment. During the experiment, participants were asked to provide their answers by button-press in a two-alternative forced choice paradigm. Response options were “shorter” or “longer”. In all experiments, each block consisted of seven repetitions of each duration test (six) leading to 56 trials per experimental condition. The entire experiment lasted ∼1 hour for a total of 448 trials (56 trials×8 blocks). The experiment was self-paced and participants were given a break between each block.

### Data Analysis

For each condition and each participant, data were averaged per trial type for each target duration leading to individual psychometric curves. Each individual curves was fitted to a normal cumulative distribution function using a non-linear least-square data fitting procedure (nlnfitDVB function) in Matlab™ (The Mathworks, Inc., Natick, MA.) An individual's point-of-subjective-equality (PSE) was determined at the 50% crossing point and the slope values estimated between the 25% and 75% crossing point. All subsequent statistical analyses including repeated measures ANOVA and paired-samples t-tests were performed using SPSS (SPSS, Inc, Chicago, IL.) Two indices were used for the estimation of the effect sizes. Cohen's d was computed as follows: 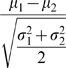, where *μ*_1_and *μ*_2_ designate the means, and 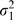 and 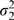 designate the variance of the control and test groups, respectively. Hedges's ĝ indices were also determined in order to provide a more conservative estimate of size effect by incorporating the sample size. Hedges's ĝ indices were computed as follows: 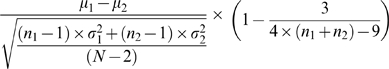, where *μ*_1_ and *μ*_2_ designate the means, 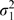 and 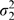 the variance, and *n*_1_and *n*_2_ the standard deviation sample size of the control and test data, respectively. N corresponds to the total number of samples.

### Bayesian Fits

The variance of the psychometric fits used to evaluate participants' PSE in each experimental condition (test and control) was extracted to compute the sensory estimates. The auditory and visual weights were computed as follows for the control conditions: 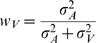 and 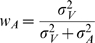, where 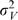 and 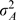 designate the variance of the visual and auditory condition, respectively. The predicted PSE were computed as the sum of the weighted unisensory PSE observed (obs) in each condition and each individual leading to the estimated (est) PSE as: 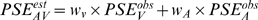. The observed and estimated PSE in AV conditions were then submitted to a paired t-test reported in Figure 4.

## Supporting Information

Figure S1 Schematic representation of auditory-visual interactions from the perspective of the ‘internal clock models’. In all the depicted internal clock models, the main components are: a pacemaker (‘tick-counter’), a switch modulated by attention, an accumulator which forwards the accumulated ticks in storage and in reference memory. The two memory components form the comparative stage between internalized duration template and test duration. The major differences between these models consist in the stage at which auditory and visual inputs converge. In the model depicted in panel a, the entire clock is ‘amodal’ in that the very first stage of time keeping (i.e. the pacemaker) do not distinguish between auditory or visual temporal cues. In the second model (panel b), the pacemaker is also shared between the two sensory modalities but the effects of attention remain separate permitting a semi-independent evaluation of the two sensory channels (note that attention can be switched between the two). In the ‘modal’ model (panel c), auditory and visual time-keeping remains independent (again, with the exception of the attentional switch) up to the amodal comparative stage.
(0.21 MB TIF)

Figure S2 Samples of fitted psychometric curves. We provide examples of the fitted psychometric for three participants tested in the Loom (top row), Recede (middle row) and Reverse (bottom row) experiments for the auditory (blue, left column), visual (green, middle column) and multisensory (red, right column) conditions. The actual data are reported as filled disc for the Test conditions and as crosses for the Control conditions. The fits are continuous lines for the Test conditions and dotted lines for the Control conditions.
(0.25 MB TIF)

Figure S3 Forced-fusion model: comparison between predicted and observed variances. In the multisensory conditions (left column), the forced-fusion predictions (black) of variance did not significantly differ from the observed variances (red) in the test (AV) and control (AVc) conditions to the exception of the AV control of the Loom experiment. Note however that the predicted variance tend to be smaller than the observed variance. To the opposite in the intersensory conditions (right column), all but one observed condition (red, Reverse visual intersensory) significantly differ from the predicted variances of the forced-fusion model (black). In particular, the observed variances are always higher than the predicted ones, suggesting the intervention of parameters not accounted for by this model. Bars indicate standard-errors of the mean.
(0.16 MB TIF)
