# Supplementary figures and images for: Distortions of Subjective Time Perception Within and Across Senses
Source: PLoS One. 2008 Jan 16;3(1):e1437. doi: 10.1371/journal.pone.0001437 (PMC2174530; doi:10.1371/journal.pone.0001437)

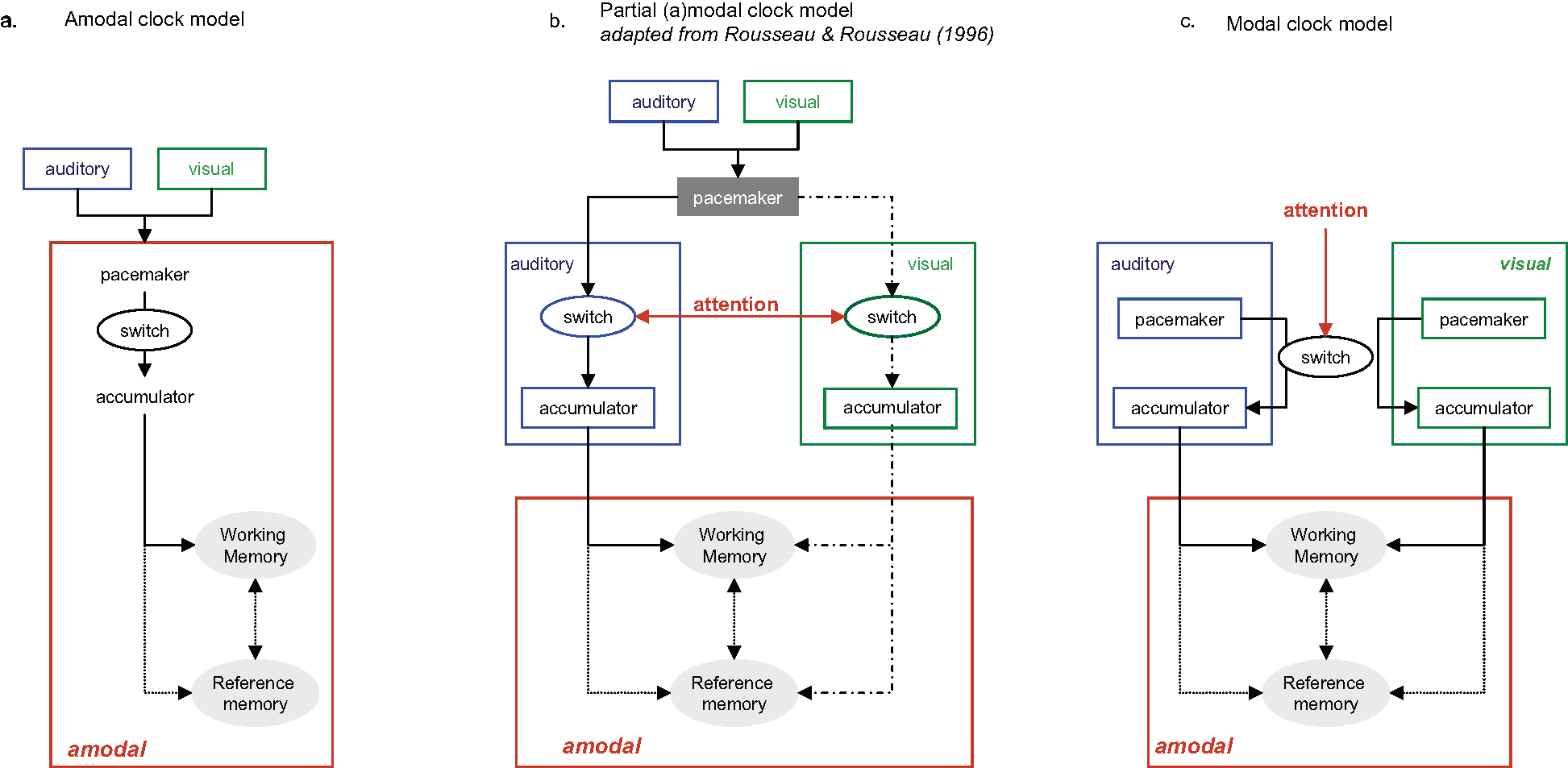

Supplement: Figure S1 — Schematic representation of auditory-visual interactions from the perspective of the ‘internal clock models’. In all the depicted internal clock models, the main components are: a pacemaker (‘tick-counter’), a switch modulated by attention, an accumulator which forwards the accumulated ticks in storage and in reference memory. The two memory components form the comparative stage between internalized duration template and test duration. The major differences between these models consist in the stage at which auditory and visual inputs converge. In the model depicted in panel a, the entire clock is ‘amodal’ in that the very first stage of time keeping (i.e. the pacemaker) do not distinguish between auditory or visual temporal cues. In the second model (panel b), the pacemaker is also shared between the two sensory modalities but the effects of attention remain separate permitting a semi-independent evaluation of the two sensory channels (note that attention can be switched between the two). In the ‘modal’ model (panel c), auditory and visual time-keeping remains independent (again, with the exception of the attentional switch) up to the amodal comparative stage. (0.21 MB TIF) [file pone.0001437.s001.tif]

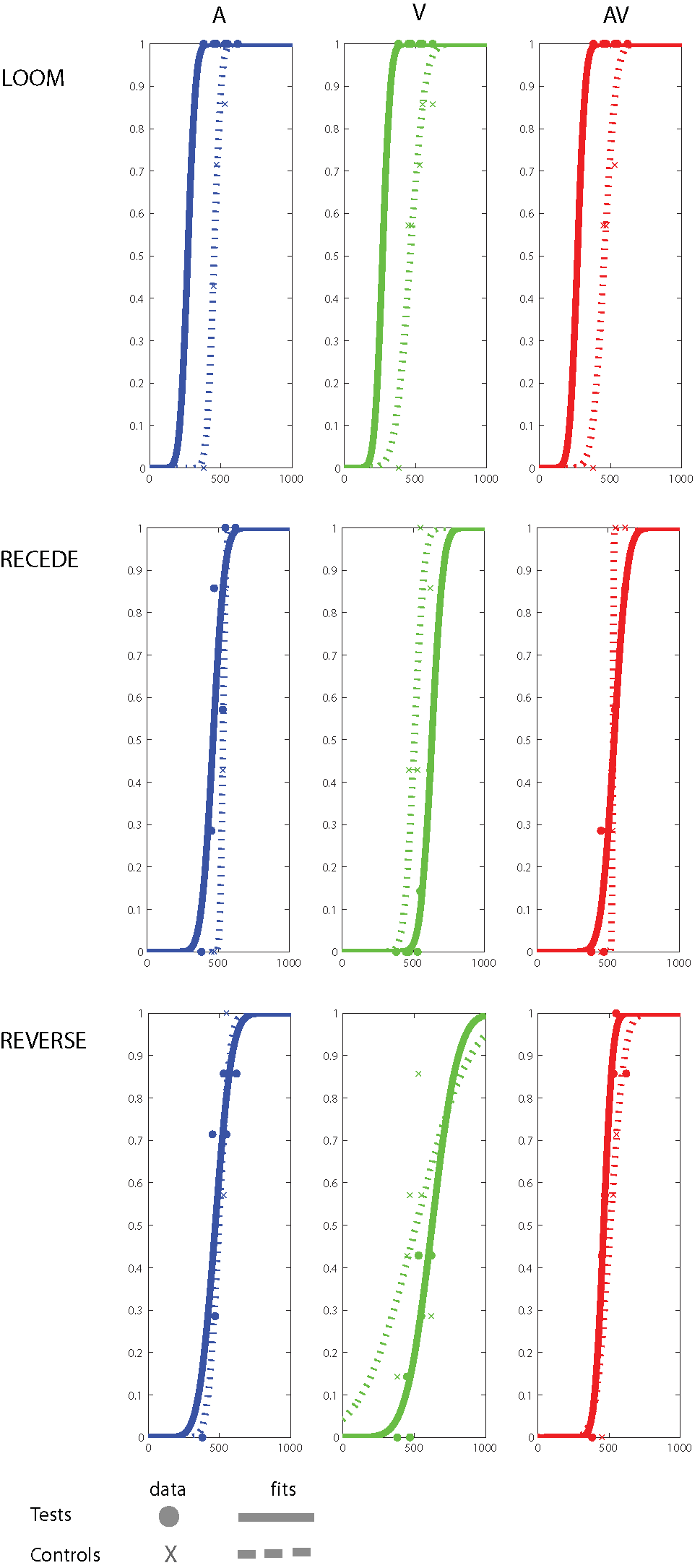

Supplement: Figure S2 — Samples of fitted psychometric curves. We provide examples of the fitted psychometric for three participants tested in the Loom (top row), Recede (middle row) and Reverse (bottom row) experiments for the auditory (blue, left column), visual (green, middle column) and multisensory (red, right column) conditions. The actual data are reported as filled disc for the Test conditions and as crosses for the Control conditions. The fits are continuous lines for the Test conditions and dotted lines for the Control conditions. (0.25 MB TIF) [file pone.0001437.s002.tif]

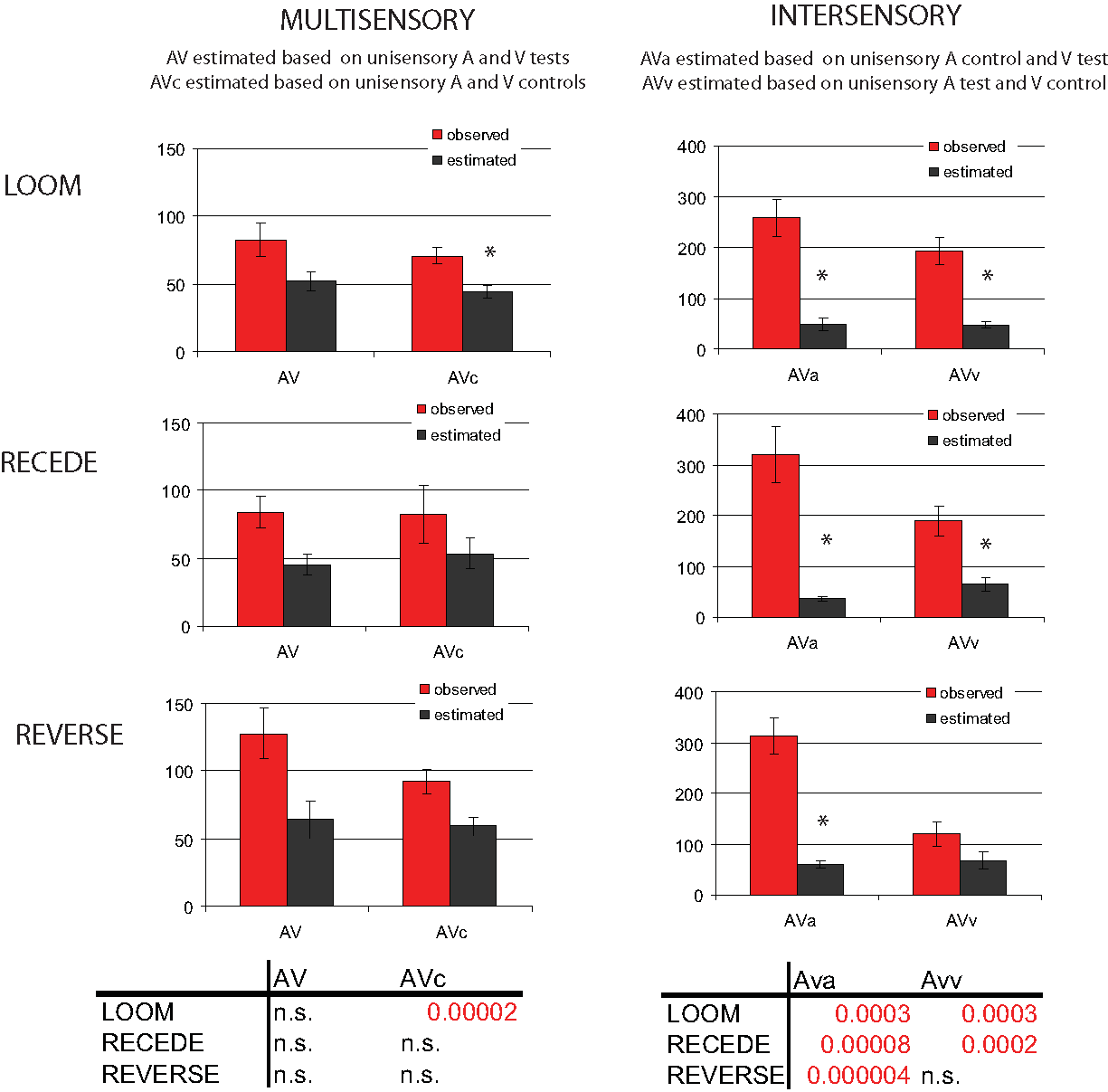

Supplement: Figure S3 — Forced-fusion model: comparison between predicted and observed variances. In the multisensory conditions (left column), the forced-fusion predictions (black) of variance did not significantly differ from the observed variances (red) in the test (AV) and control (AVc) conditions to the exception of the AV control of the Loom experiment. Note however that the predicted variance tend to be smaller than the observed variance. To the opposite in the intersensory conditions (right column), all but one observed condition (red, Reverse visual intersensory) significantly differ from the predicted variances of the forced-fusion model (black). In particular, the observed variances are always higher than the predicted ones, suggesting the intervention of parameters not accounted for by this model. Bars indicate standard-errors of the mean. (0.16 MB TIF) [file pone.0001437.s003.tif]
